# A Machine Learning Framework for Diagnosing and Predicting the Severity of Coronary Artery Disease

**DOI:** 10.31083/j.rcm2406168

**Published:** 2023-06-08

**Authors:** Aikeliyaer Ainiwaer, Wen Qing Hou, Kaisaierjiang Kadier, Rena Rehemuding, Peng Fei Liu, Halimulati Maimaiti, Lian Qin, Xiang Ma, Jian Guo Dai

**Affiliations:** ^1^Department of Cardiology, The First Affiliated Hospital of Xinjiang Medical University, 830011 Urumqi, Xinjiang, China; ^2^College of Information Science and Technology, Shihezi University, 832003 Shihezi, Xinjiang, China

**Keywords:** machine learning, coronary artery disease, SYNTAX score, GENSINI score

## Abstract

**Background:**

Although machine learning (ML)-based prediction of coronary artery disease (CAD) has gained increasing attention, assessment of the severity of suspected CAD in symptomatic patients remains challenging.

**Methods:**

The training set for this study consisted of 284 retrospective participants, while the test set included 116 prospectively enrolled participants from whom we collected 53 baseline variables and coronary angiography results. The data was pre-processed with outlier processing and One-Hot coding. In the first stage, we constructed a ML model that used baseline information to predict the presence of CAD with a dichotomous model. In the second stage, baseline information was used to construct ML regression models for predicting the severity of CAD. The non-CAD population was included, and two different scores were used as output variables. Finally, statistical analysis and SHAP plot visualization methods were employed to explore the relationship between baseline information and CAD.

**Results:**

The study included 269 CAD patients and 131 healthy controls. The eXtreme Gradient Boosting (XGBoost) model exhibited the best performance amongst the different models for predicting CAD, with an area under the receiver operating characteristic curve of 0.728 (95% CI 0.623–0.824). The main correlates were left ventricular ejection fraction, homocysteine, and hemoglobin (*p* < 0.001). The XGBoost model performed best for predicting the SYNTAX score, with the main correlates being brain natriuretic peptide (BNP), left ventricular ejection fraction, and glycated hemoglobin (*p* < 0.001). The main relevant features in the model predictive for the GENSINI score were BNP, high density lipoprotein, and homocysteine (*p* < 0.001).

**Conclusions:**

This data-driven approach provides a foundation for the risk stratification and severity assessment of CAD.

**Clinical Trial Registration:**

The study was registered in www.clinicaltrials.gov protocol registration system (number NCT05018715).

## 1. Introduction 

Artificial intelligence is an important tool in the current era of big data and can improve human productivity by simulating human learning thought processes and analyzing complex data [1]. Currently, machine learning (ML) and a subset of ML, deep learning, are the most common methods used in artificial intelligence [2]. The inception of machine learning (ML) can be traced back to the 1950s and 1960s [2], when scholars commenced investigating the plausibility of employing computers for self-regulating learning and discerning decision-making, accomplished through the construction of mathematical models and algorithms [3]. This approach empowers computers to continually enhance and optimize their functioning by processing and learning from data [4]. ML deviates from traditional rule-based programming by placing a greater emphasis on automatic pattern recognition in data, thereby precluding the need for manual rule design. Deep learning is commonly used to analyze raw clinical data and imaging data [4], while ML can be used to predict the severity and prognosis of cardiovascular disease [5]. Artificial intelligence is now commonly used in medicine and has been advancing progressively in the cardiovascular field [6].

The diagnosis of coronary artery disease (CAD) and early intervention in symptomatic patients with suspected CAD is challenging [7], and its definitive diagnosis in clinical practice remains complicated [8]. Although current methods reduce the probability of misdiagnosis of stable CAD, the invasive diagnostic procedures used can be considered an overly medical approach. Therefore, the development of a scoring system that accurately predicts coronary artery stenosis in patients suspected of CAD and its severity could reduce the number of downstream and invasive diagnostic tests [9]. Thus far, investigators have proposed multiple testing strategies to effectively screen patients with suspected CAD, the most notable being the Diamond Forrest model. However, research suggests this model has a high false positive rate. As a result, a “battle of the scores” has ensued over the past decade for predicting the pretest probability of coronary heart disease. Many “up-to-date” risk assessment models have emerging based on the latest clinical trial data. However, these methods still cannot accurately assess the complications of CAD and hence their application in clinical practice remains limited [8].

The GENSINI score reflects plaque loading, but not bifurcation, calcification and tortuous lesion characteristics. The SYNTAX score on the other hand reflects the type of plaque and the complexity of percutaneous coronary intervention (PCI). It also describes the anatomy of the coronary lesion and provides guidance to clinicians when developing optimal treatment plans for high-risk patients. The SYNTAX score can help with making treatment decisions for patients with lesions suitable for both PCI and coronary artery bypass graft (CABG) and in whom the surgical mortality rate is expected to be low.

The goal of this study was therefore to develop a ML model based on the clinical characteristics of a retrospective cohort comprising CAD patients and healthy controls. The model was then tested in a prospective cohort. The objectives of the study were first to use ML and statistical methods to identify new risk factors associated with disease severity in CAD, and second to develop an electronic medical record- and coronary score-driven ML model that was predictive for the detection of severe CAD.

## 2. Materials and Methods

### 2.1 Methods

A three-step modeling procedure was used to achieve the research goals [10]. In the first, patients were divided into two groups based on coronary angiographic findings: a coronary group (stenosis ≥50) and a non-coronary group (stenosis <50%, or no stenosis) [11, 12]. The second step was to provide estimates of the SYNTAX and GENSINI scores for patients undergoing coronary angiography [13]. In the third step, 53 clinical characteristics were used as input to predict diagnosis (For example, sex, age, BMI, etc.), the GENSINI and SYNTAX scores (Table 1) [14]. Feature selection deep learning techniques were also used and these provide a way to identify potential risk factors for CAD based on ML. This allows a better understanding of the medical and clinical features associated with the presence or absence of CAD, with the outcome derived from the SYNTAX score distribution, and with the outcome derived from the GENSINI score. The methodology to be evaluated is designed to provide a uniform risk score that can help to determine the need for invasive or functional non-invasive tests in patients with suspected CAD, as well as for patients with complex CAD who need more rigorous coronary revascularization surgery. The development of an automated recommendation system based on data-driven, perspective analysis ML algorithms should thus provide an auxiliary means for personalized treatment in routine clinical practice.

**Table 1.** **Machine learning model input and output characteristics**.

Feature type	In put features	Binary classification algorithm	Out put	Regression algorithms	Out put
General information	Age, gender, education, nation, diastolic blood pressure, systolic blood pressure, body mass index, drinking history, smoking history, pulse rate	1. SVM 2. XGB 3. RF 4. NB 5. LR 6. GBC 7. Adaboots	Predicting subjects with or without coronary artery disease	1. XGB 2. Decision Tree 3. linear 4. SVM 5. K-Neighbors 6. RF 7. Ada-boost 8. Bagging 9. Extra-Tree	Predicting subjects’ SYNTAX score & Predicting subjects’ GENSINI score
Medical history	Symptom, previous history (hypertension, type 2 diabetes, hyperlipidemia, coronary heart disease, chronic renal failure), family history (hypertension, type 2 diabetes, hyperlipidemia, coronary heart disease, chronic renal failure), Medication History (antiplatelet drugs, statins, angiotensin receptor blockers, angiotensin converting enzyme inhibitor drugs, calcium channel blocker, beta blocker, diuretics, nitrates, glucose-lowering drugs), history of drug allergy, surgical history				
Laboratory examination	Troponin I, creatine-phosphokinase, isoenzymes Myoglobin, brain natriuretic peptide, Leukocytes, Hemoglobin, $K^{+}$, ${\mathrm{Na}}^{+}$, ${\mathrm{Cl}}^{-}$, Blood glucose, Triglycerides, Total cholesterol, high density lipoprotein, low density lipoprotein, C-reactive protein, interleukin 6, Calcitoninogen, D dimer, Homocysteine, Glycated hemoglobin				
Imaging examination	Ventricular wall motion abnormalities, ejection fraction%				

SVM, Support Vector Machine; XGB, eXtreme Gradient Boosting; RF, Random Forest; NB, Naive Bayes; LR, Logistic Regression; GBC, Gradient Boosting Classifier; Adaboots, Adaptive Boosting; K-Neighbors, K-Nearest Neighbors Regression.

Unlike previous studies [8], the present investigation included a population with <50% coronary stenosis for regression analysis. There were two reasons for this. First, a significant proportion of patients in our study cohort had coronary stenosis in the 0–50% range, but exhibited clear symptoms of CAD. Recent research on this population suggests that disease progression without early intervention can have serious consequences. These patients were therefore included with the aim of guiding physicians in the development of protocols for early coronary prevention. Second, this population was also an accurate representation of the real-world population, thus making it easier to reproduce in future work. SYNTAX scores were obtained using online evaluation on the website (http://www.syntaxscore.org/). The GENSINI score is based on coronary angiographic findings and was calculated by multiplying the stenosis score at the site of the lesion by the appropriate weighting factor. The sum of all the lesion branch scores is the GENSINI score [15].

This study included patients who underwent elective or urgent coronary angiography at the First Affiliated Hospital of Xinjiang Medical University. We attempted to develop new risk prediction algorithms for CAD-related risk factors and for CAD severity using clinical indicators in combination with coronary angiographic features and with two different scoring criteria.

### 2.2 Participants

The training set consisted of data from 284 retrospective participants, while the test set was comprised of 116 prospectively enrolled participants [16]. Patients were eligible for the test set only if they were judged to have stable angina. The exclusion criteria were a previous diagnosis of CA, previous diagnosis of acute coronary syndrome (ACS), previous history of PCI or CABG, severe infection, or renal or pulmonary comorbidities [17].

### 2.3 Model Building Process

**Fig. 1.**
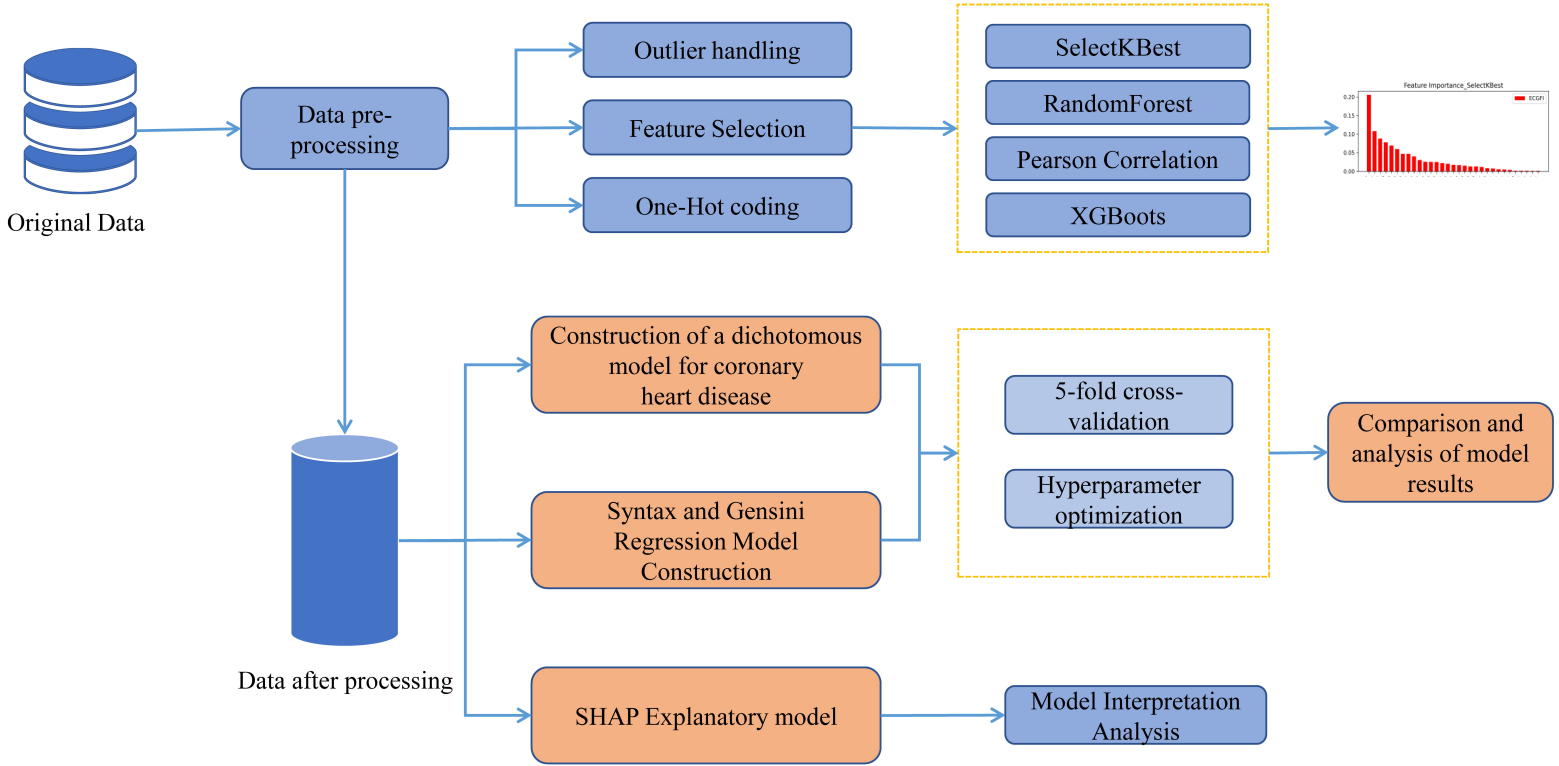
**The research process is depicted in the diagram where the raw data is initially subjected to pre-processing and fed into distinct regression and classification algorithms**. Following the model training and hyperparameter tuning, the ultimate prediction outcomes are generated, and the SHAP framework is employed for model interpretation.

A three-step approach was used for building the model: database creation, model construction, and model interpretation and evaluation.The detailed technical path is shown in Fig. 1.

#### 2.3.1 Database Creation

In the first step, each patient’s medical data was collected from electronic medical records. The SYNTAX and GENSINI scores for each patient were independently assessed by two cardiologists. Disagreements in the coronary evaluation were assessed by a third specialist who then made the final decision.

All of the original data were summarized and stored, and then carefully checked to ensure they met the quality standards for the tasks performed [18]. To this end, descriptive statistical methods and visualization techniques were used to summarize patient characteristics for assessment by the cardiologists and to identify features that are meaningful for the construction of ML models [19].

#### 2.3.2 Data Processing and Feature Selection

The original dataset contained 53 feature attributes. These were initially processed using the Pandas package in Python to convert the raw data into Int and Float types that could be used for ML operations [20]. The Filter and Embedded methods were applied for analysis of the clinical features [21]. The Filter method primarily employs techniques such as the chi-square test and correlation coefficient, whereas the Embedded method integrates feature selection into ML algorithms to identify the most relevant features through the learning process. Notably, the extreme gradient boosting (XGBoost) and random forest (RF) algorithms are the most relevant approaches in this context [22, 23]. The XGBoost algorithm is well-suited for the processing of clinical data [22, 24], while the RF algorithm has the advantages of high accuracy in feature selection, avoidance of overfitting, and broad applicability [23]. In view of the dimensionality and feature relevance of the dataset, we chose to use the XGBoost regressor and RF regressor function packages to filter the clinical features [25]. Ultimately, the algorithm that considers the area under the receiver operating characteristic (ROC) curve to be the largest is the best algorithm for constructing the dataset by comparing the ML feature filtering performance [26].

#### 2.3.3 Model Building

The building phase for our experimental model consisted of two steps. In the first, the binary classification problem is addressed, with the model built after labeling patients as either “diseased” or “disease-free” based on their coronary angiography results [11, 27].

During the model training process, regularization techniques and weight adjustment of samples were employed to enhance the model prediction ability, given the limited sample size and the unbalanced categories in the dataset. Furthermore, a 5-fold cross-validation was used for model selection (**Supplementary Fig. 1**) [28], as well as hyperparameter adjustment to prevent overfitting and to improve model generalization. Specifically, L1 and L2 regularization techniques were used to select important features, to reduce the weighting of unimportant features, and to avoid overfitting. Sample-based weight adjustment was also used to balance the dataset by assigning higher weights to minority categories of samples [29]. This drives the model to assign higher weights to minority categories during training [30]. Sample weights were determined by calculating the ratio of the weights of positive samples (representing the minority categories) to the weights of negative samples (representing the majority categories). More specifically, this ratio was calculated as the number of samples in the majority category divided by the number of samples in the minority category. Furthermore, 5-fold cross-validation was used for model selection and for hyperparameter adjustment to prevent overfitting and to improve the ability for model generalization. The model parameters are shown in **Supplementary Table 1**.

Following the completion of training on the training dataset, the model was tested on the test dataset to validate the performance metrics [30]. The second step in the model building involved a regression analysis and was modeled based on the SYNTAX and GENSINI scores. From the large number of candidate models available for modeling classification and regression, a total of 7 dichotomous classification models and 9 regression models were selected [31]. The input and output for these models are described in detail in Table 1.

#### 2.3.4 Model Interpretation and Evaluation

To address the challenge of limited model interpretability, the SHAP framework was incorporated to provide an explanation of the model outcomes, thereby increasing confidence in the results. The SHAP value quantifies the extent to which each feature in the model contributes to the prediction. It also facilitates with visualization of the results.

For the evaluation of performance, various metrics have been employed to evaluate the efficacy of ML models in both classification and regression tasks. For dichotomous models, a range of evaluation metrics were employed across five dimensions, including area under the ROC curve, R-squared, accuracy, precision, recall, and F1-score [32]. Performance evaluation was carried out by calculating the mean squared error, mean absolute error (MAE), mean absolute prediction error, and coefficient of determination (R2) [33], as detailed below.

$$\text{ Accuracy }=\frac{T\,P+T\,N}{N}$$

$$\text{ Precision }=\frac{T\,P}{T\,P+F\,P}$$

$$\text{ Recall }=\frac{T\,P}{T\,P+F\,N}$$

$$\left\{ \begin{array}{c} F\,1_{-}\,S\,c\,o\,r\,e_{i}=\frac{2\times \text{ Precision }\times \text{ Recall }}{\text{ Precision }+\text{ Recall }}\\ F\,1\,\_\,S\,c\,o\,r\,e=\frac{n_{i}}{A}\,\sum_{i=1}^{n}F\,1\,\_\,S\,c\,o\,r\,e_{i} \end{array}\right.$$

$$R^{2}=1-\frac{\sum_{i}{\left(\hat{y_{i}}-y_{i}\right)}^{2}}{\sum_{i}{\left(\bar{y}-y_{i}\right)}^{2}}$$

$$\mathrm{MAE}=\frac{1}{N}\,\sum_{i=1}^{N}\left|y_{i}-{\hat{y}}_{l}\right|$$

$$\mathrm{MSE}=\frac{1}{N}\,\sum_{i=1}^{N}{\left(y_{i}-{\hat{y}}_{l}\right)}^{2}$$

Where N denotes the total number of samples tested, TP (True Positive) denotes a true case, TN (True Negative) denotes a true negative case, FP (False Positive) denotes a false positive case, and FN (False Negative) denotes a false negative case. y_i denotes the true value of the i-th sample, (y_i )^ denotes the predicted value of the i-th sample, and $\bar{y}$ denotes the mean of the true values of all samples.

The ML framework proposed in this study was implemented in the python programming language. Differences were considered statistically significant when two-sided tests showed a *p*-value < 0.05.* p*-values were corrected for multiple testing using the Benjamini-Hochberg procedure [34]. All tests were two-tailed (non-directional), i.e., the alternative hypothesis was that the indicators being measured were not equal.

## 3. Results

### 3.1 Predictive Factors

Patients with well-established CAD risk factors, such as hypertension, type-2 diabetes, smoking and alcohol consumption, were generally found to have higher coronary vascular score values than those without (Table 2). Indeed, non-parametric Mann‒Whitney tests showed that hypertension (*p* = 0.002, *p* = 0.002), type-2 diabetes (*p* < 0.001, *p* < 0.001), and smoking (*p* = 0.028, *p* = 0.007) had statistically significant effects on the distribution of SYNTAX scores, and GENSINI scores. Alcohol consumption had no significant effect on the distribution of the two scores (*p* = 0.307, *p* = 0.160), but a significant effect on diagnosis (*p* = 0.003). For the persistent risk factors (Table 3), non-parametric Spearman’s rho test showed significant positive correlations between age, troponin, creatine-phosphokinase (CPK), myoglobin (MB), brain natriuretic peptide (BNP), glucose (Glu), interleukin 6, D-dimer, homocysteine (Hcy) and glycosylated hemoglobin (GHb) levels, and diagnosis, SYNTAX score, and GENSINI score (r >0, *p* < 0.05). total cholesterol (TC), high- density lipoprotein (HDL), low- density lipoprotein (LDL), ejection fraction (EF%) values and SYNTAX score response negative correlation (r <0, *p* < 0.05).

**Table 2.** **Descriptive and exploratory analyses for categorical risk factors and scores**.

			Diagnosis	SYNTAX		GENSINI	
Factor		N	*p*a	Median (P25, P75)	*p*b	Median (P25, P75)	*p*c
Sex			<0.001		<0.001		<0.001
	Male	267 (66.8%)		9 (2, 19.5)		29 (4, 84)	
	Female	133 (33.3%)		0 (0, 7)		4 (0, 15)	
Hypertension			<0.001		0.002		0.002
	NO	230 (57.5)		2 (0, 12)		7 (0, 50.5)	
	YES	170 (42.5)		9 (2, 18)		28.5 (5, 84.5)	
Type 2 diabetes (T2D)			<0.001		<0.001		<0.001
	NO	309 (77.3)		5 (0, 13.25)		10 (0, 56)	
	YES	91 (22.8)		14 (2, 21.5)		42 (8, 98)	
Smoking			0.004		0.028		0.007
	NO	276 (69)		5 (0, 14)		10 (1, 57.5)	
	YES	124 (31)		9 (2, 19)		30 (4, 83.5)	
Alcohol consumption			0.003		0.307		0.160
	NO	344 (83.5)		5 (0, 15.5)		12 (2, 70)	
	YES	66 (16.5)		6.5 (2, 18.25)		27.5 (4, 88)	
Antiplatelet drugs			<0.001		0.027		0.047
	NO	282 (70.5)		5 (0, 14)		9 (0.75, 65)	
	YES	118 (29.5)		9 (2, 19)		34 (6.5, 75.5)	
ARBs			0.005		0.006		0.011
	NO	349 (87.3)		5 (0, 14)		10 (2, 66.5)	
	YES	51 (12.8)		12 (5, 20)		44.5 (12, 97)	
Statins			0.004		0.335		0.751
	NO	293 (73.3)		5 (0, 14.5)		10 (2, 70.5)	
	YES	107 (26.8)		7 (2, 19)		24 (4, 73)	
CCBs			0.002		0.064		0.126
	NO	314 (78.5)		5 (0, 15)		10 (2, 68)	
	YES	86 (21.5)		11 (2, 17)		30 (4, 78.25)	
Glucose lowering drugs			0.002		0.002		0.001
	NO	317 (79.3)		5 (0, 14)		10 (5, 59)	
	YES	83 (20.8)		12 (2, 21)		36 (8, 98)	
LVWMAs			<0.001		<0.001		<0.001
	NO	313 (78.3)		2 (0, 12)		9 (0, 43)	
	YES	87 (21.8)		16.5 (5, 26)		70 (13, 115)	

*p*a, *p* value of diagnosis p erformance; *p*b, *p* value of SYNTAX score; *p*c, *p* value of GENSINI score; LVWMAs, Left ventricular wall motion abnormalities; ARBs, angiotensin receptor blockers; CCBs, calcium channel blockers. Median (P25, P75), the median (25th percentile–75th percentile), we correct these *p*-values for multiple testing.

**Table 3.** **Descriptive and exploratory analyses for continuous risk factors and scores**.

Risk factor	M	Mdn	SD	Min	Max	Diagnosis (*p*)	SYNTAX (*p*)	GENSINI (*p*)
Age	58.962	58.000	11.112	28	91	<0.001	<0.001	<0.001
BMI	25.829	25.000	3.613	17	42	0.085	0.087	0.045
SBP	128.000	126.000	17.598	88	187	0.676	0.490	0.244
DBP	77.060	77.000	11.336	47	124	0.265	0.572	0.754
TnI	0.092	0.012	0.518	0.012	8.310	0.603	<0.001	<0.001
Pulse	77.950	76.000	10.049	55	129	0.283	0.572	0.984
CPK	0.977	0.740	0.810	0.220	5.52	0.017	<0.001	<0.001
MB	34.893	28.745	23.694	10.130	179	0.017	<0.001	<0.001
BNP	311.850	90.200	718.036	10.00	6120	0.003	<0.001	<0.001
Leukocytes	6.930	6.685	1.784	3.490	16	0.253	0.016	0.046
Hemoglobin	142.090	142.000	14.598	95	206	0.603	0.897	0.893
$K^{+}$	3.8062	3.780	0.346	2.910	5	0.345	0.662	0.630
${\mathrm{Na}}^{+}$	141.5183	141.640	2.684	126.560	149	0.772	0.623	0.567
${\mathrm{Cl}}^{-}$	105.721	105.850	2.944	96.700	114	0.603	0.495	0.520
Glu	7.126	6.130	3.218	2.730	25	0.058	0.001	<0.001
TG	1.955	1.730	1.056	0.480	8	0.891	0.825	0.910
TC	4.089	4.040	1.070	1.660	8	<0.001	<0.001	<0.001
HDL	1.007	0.970	0.292	0.450	3	<0.001	<0.001	<0.001
LDL	2.4322	2.350	0.866	0.680	5	<0.001	<0.001	<0.001
CRP	8.129	6.100	7.362	5.000	90	0.283	0.032	0.009
IL6	4.241	2.555	6.671	1.500	74	0.009	<0.001	<0.001
CT	0.035	0.030	0.047	0.020	0.820	0.322	0.002	0.028
Ddimer	131.584	85.000	198.430	0.600	2993	0.017	<0.001	<0.001
Hcy	12.676	11.525	5.434	5.800	59	<0.001	<0.001	<0.001
GHb	6.374	6.000	1.359	4.300	13	<0.001	<0.001	<0.001
EF(%)	60.922	62.680	6.155	30.720	70	<0.001	<0.001	<0.001

BMI, Body Mass Index; SBP, systolic blood pressure; DBP, diastolic blood pressure; TnI, Troponin I; CPK, creatine-phosphokinase; MB, myoglobin; BNP, brain natriuretic peptide; Glu, glucose; TG, Triglyceride; TC, Total cholesterol; HDL, high density lipoprotein; LDL, low-density lipoprotein; CRP, C Reactive Protein; IL6, Interleukin 6; CT, clotting time; Hcy, homocysteine; GHb, Glycosylated Hemoglobin; EF%, ejection fraction; Mdn, median; SD, standard deviation. We correct these *p*-values for multiple testing.

### 3.2 Visualization Analysis

**Fig. 2.**
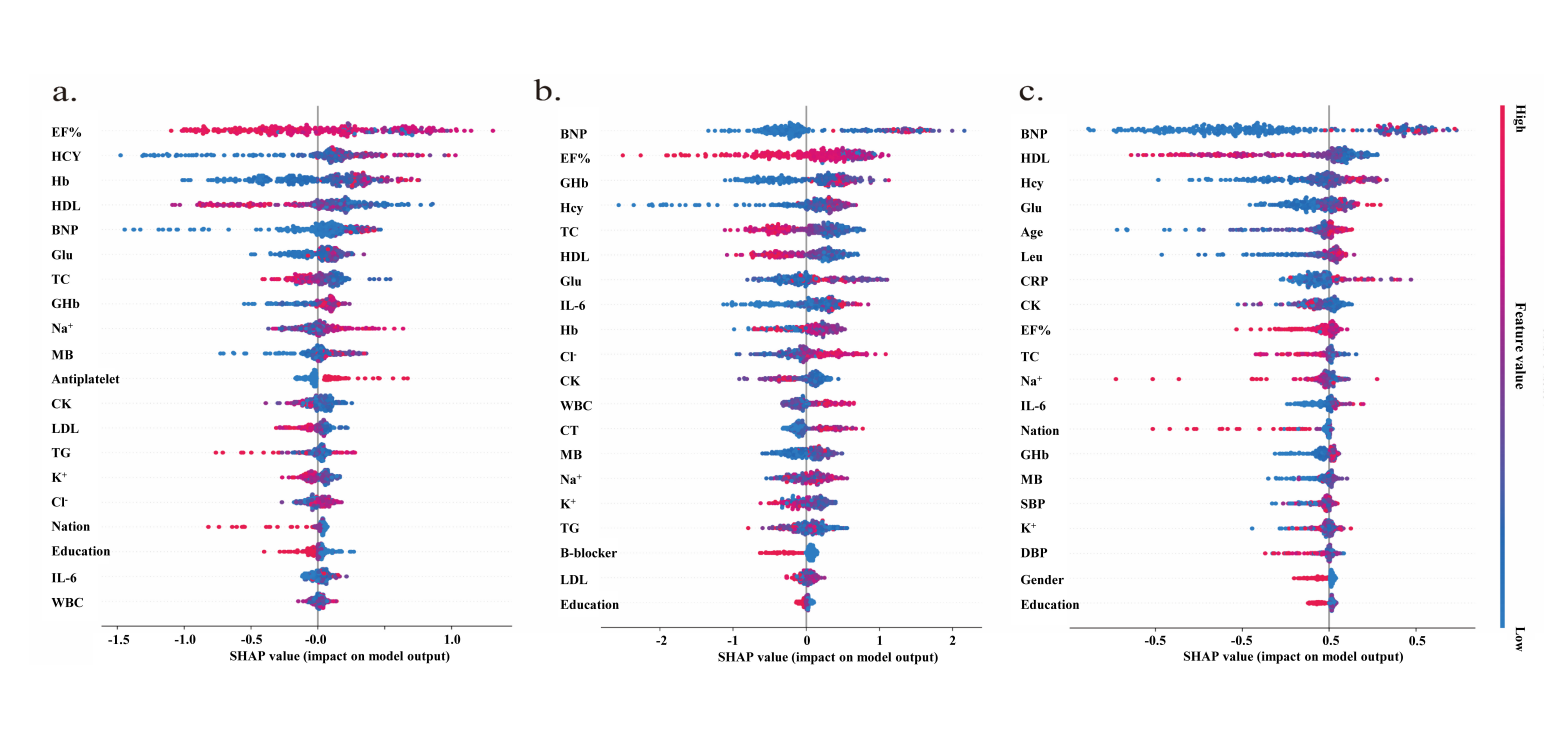
**Results of screening clinical features using machine learning algorithms**. (a) Distribution of Shapley values for the screened clinical features of the best performing diagnostic model. (b) Distribution of Shapley values for the screened clinical features of the best-performing model based on SYNTAX score. (c) Distribution of Shapley values for the screened clinical features of the best-performing model based on the GENSINI score. EF%, ejection fraction; Hcy, homocysteine; Hb, hemoglobin; HDL, high density lipoprotein; BNP, brain natriuretic peptide; Glu, glucose; TC, total cholesterol; GHb, glycosylated hemoglobin; MB, myoglobin; CK, creatine Kinase; TG, triglyceride; LDL, low-density lipoprotein; IL6, interleukin 6; WBC, white blood cell; β-blocker, Beta blockers; CRP, C reactive protein; Leu, leucocyte; SBP, systolic blood pressure; DBP, diastolic blood pressure.

After screening for key features that affect diagnostic and regression models, SHAP visualization analysis was performed in two separate parts. Fig. 2 summarizes the risk factors that had a significant impact on diagnosis and on the SYNTAX and GENSINI scores. Left ventricular ejection fraction, homocysteine, hemoglobin, HDL, and BNP each had a significant effect in the diagnostic model. BNP, EF%, MB, GHb, and TC were important features in the regression models for accurate prediction by the SYNTAX score, while BNP, HDL, GHb, glucose, and age were important for accurate prediction by the GENSINI score.

**Fig. 3.**
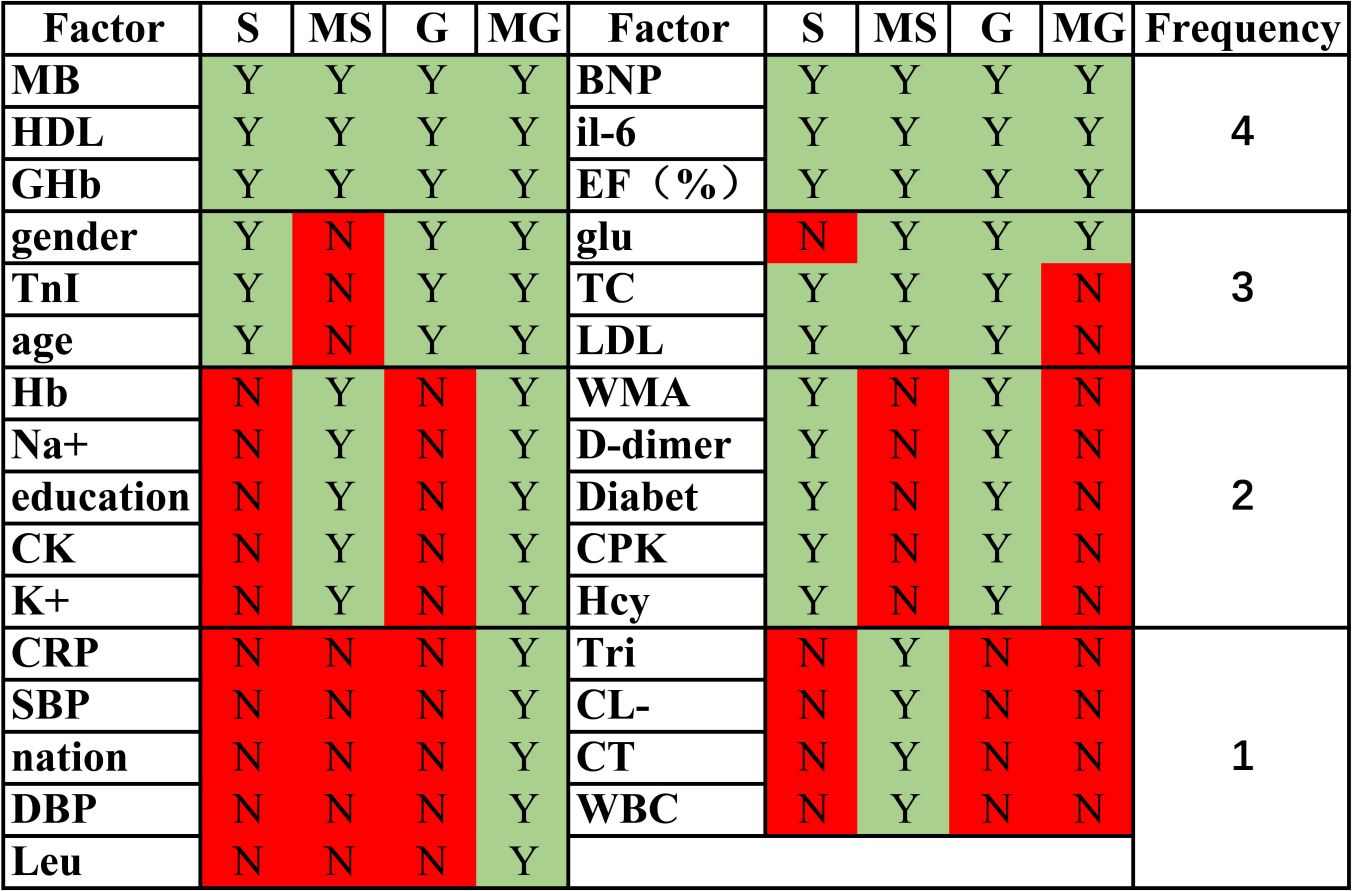
**Similarities and differences in correlation factors in regression models and statistical analysis**. S: SYNTAX score; MS: machine learning SYNTAX score; G: GENSINI score; MG: machine learning GENSINI score. The frequency represents the number oftimes that factor was considered to have an effect on the score in the S, MS, G, and MG scoring methods. EF%, ejection fraction; Hcy, homocysteine; Hb, Hemoglobin; HDL, high density lipoprotein; BNP, brain natriuretic peptide; Glu, glucose; TC, Total cholesterol; MB: myoglobin; CK: Creatine Kinase; LDL, low-density lipoprotein; IL6, Interleukin 6; WBC, white blood cell; CRP, C Reactive Protein; Leu, leucocyte; SBP, systolic blood pressure; DBP, diastolic blood pressure; TnI, Troponin I; CPK, creatine-phosphokinase; CT, clotting time; GHb, Glycosylated Hemoglobin; WMA, Left ventricular wall motion abnormalities. Green indicates statistically significant or meaningful in the machine learning models, while red indicates not meaningful.

Nine metrics outside of the statistical analysis of SYNTAX score correlations were identified in the deep learning algorithm based on SYNTAX scores (Fig. 3). These factors were not identified by assessing the Spearman correlation coefficients. In fact, statistical evaluation of rigorous SYNTAX scores found that K+ had a significant positive correlation (*p* = 0.025, r = 0.611). Education had a significant negative correlation (*p* = 0.026, r = –0.111) but the r value was close to 0, indicating only a weak correlation. In the deep learning algorithm based on GENSINI scores, 10 indicators were identified outside of the analysis of GENSINI score correlations. None of these factors was found to be significant by assessing Spearman’s correlation coefficient. Indeed, statistical evaluation of the GENSINI scores showed very weak correlations with Leu (*p* = 0.032, r = 0.107) and CRP (*p* = 0.05, r = 0.142).

### 3.3 Model Evaluation

**Fig. 4.**
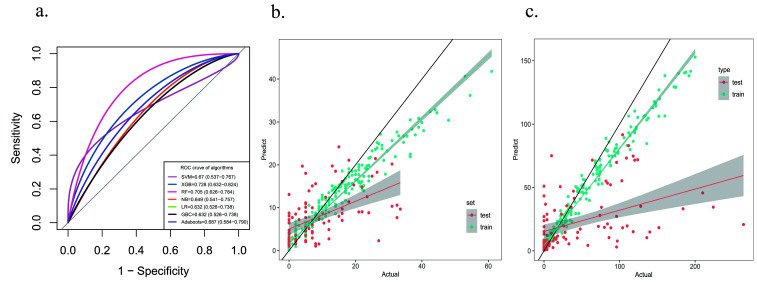
**Model evaluation**. (a) Comparison of ROC curves of diagnostic models. (b) Scatterplot of the regression model based on SYNTAX score. (c) Scatterplot of the regression model based on GENSINI score. ROC, receiver operating characteristic.

We next evaluated the performance of the classifiers and regression models, as summarized in Tables 4,5. Two specific classification models were found to have advantages. Multidimensional evaluation revealed the RF model performed best in terms of sensitivity, specificity, and recall in a balanced manner. The XGBoost classifier performed best in terms of the area under the ROC curve (Fig. 4a). For the regression models, XGBoost dominated for the prediction of SYNTAX and GENSINI scores (Fig. 4b,c). A key issue from the clinician’s perspective is whether the method can explain the results. Practical evidence suggests that BNP, EF%, lipids, age and glucose are some of the main risk factors for the development and progression of cardiovascular disease, for cardiovascular disease prognosis, and for the occurrence of adverse cardiovascular events.

**Table 4.** **Multidimensional evaluation of diagnostic models**.

Model	AUC	$R^{2}$	Accuracy	Precision	Recall	F1-score
SVM	0.670	0.532	0.653	0.653	1.000	0.790
XGB	0.728	0.203	0.727	0.745	0.886	0.809
RF	0.705	0.094	0.752	0.753	0.924	0.830
NB	0.649	0.714	0.612	0.808	0.532	0.641
LR	0.632	0.021	0.769	0.780	0.899	0.835
GBC	0.632	0.052	0.785	0.791	0.911	0.847
Adaboots	0.687	0.240	0.719	0.732	0.899	0.867

$R^{2}$, determination coefficient; AUC, Area Under the ROC Curve; SVM, Support Vector Machine; XGB, eXtreme Gradient Boosting; RF, Random Forest; NB, Naive Bayes; LR, Logistic Regression; GBC, Gradient Boosting Classifier; Adaboots, Adaptive Boosting.

**Table 5.** **Regression model evaluation**.

GENSINI-model	MAE	$R^{2}$	MSE	SYNTAX-model	MAE	$R^{2}$	MSE
XGB	4.876	0.484	56.753	XGB	4.629	0.535	51.141
Decision Tree	6.167	0.341	72.490	Decision Tree	5.834	0.155	92.968
linear	6.110	0.523	104.316	linear	5.987	0.064	103.040
SVM	5.616	0.234	84.271	SVM	5.246	0.240	83.690
K-Neighbors	5.987	0.065	102.913	K-Neighbors	5.493	0.036	113.994
Random Forest	5.395	0.415	64.359	Random Forest	6.376	0.307	76.268
Ada-boost	5.492	0.246	82.994	Ada-boost	6.425	0.305	76.468
Bagging	5.933	0.146	94.028	Bagging	5.735	0.163	92.111
Extra-Tree	6.58	0.211	86.743	Extra-Tree	5.834	0.155	92.968

MSE, mean squared error; MAE, mean absolute error; $R^{2}$, determination coefficient; SVM, Support Vector Machine; XGB, eXtreme Gradient Boosting; Ada-boots, Adaptive Boosting; K-Neighbors, K-Nearest Neighbors Regression.

## 4. Discussion

The accuracy of early coronary risk assessment during hospitalization is critical for the proper management of CAD, which requires different treatment modalities according to the level of disease severity. During the risk assessment of cardiovascular disease in routine clinical practice, clinicians tend to overly focus on laboratory indicators and non-laboratory patient characteristics such as BMI and gender are often underestimated. Although the latter are important risk factors for cardiovascular disease, they are often considered less important when assessing disease severity. Coronary angiography can be a good diagnostic tool for CAD, but has the disadvantages of being complicated to perform and prone to adverse reactions. For example, in one study ascular complications reached 11.7% and the incidence of contrast nephropathy reached 3.3% [35]. Patients are also inclined to refuse the test in the early stages of the disease. Therefore, coronary angiography is generally used to confirm the diagnosis of CAD after the onset of obvious significant symptoms. It is not used for the purpose of early screening or diagnosis, thus leading to many problems such as untimely treatment of patients and poor disease control.

In the current study we selected 53 clinical indicators and built ML models to investigate the nonlinear relationship between these indicators and the diagnostic outcome of CAD patients. Additionally, we constructed ML models with the aim of assessing the severity of CAD patients based on clinical indicators. Our findings demonstrate that ML algorithms can be used to predict the risk of coronary heart disease, thereby assisting physicians to diagnose the disease more accurately. We evaluated multiple models to compare the efficacy of different ML algorithms. The results showed that integrated learning outperformed other methods of diagnosing coronary heart disease by combining the results of multiple classifiers. In particular, the XGBoost [36] model identified the top 15 indicators important for disease prediction (EF%, BNP, HCY, etc.), with an accuracy >90%. We found that XGBoost is well-suited for typical structured data such as tabular and time series data, and can be used for both classification and regression tasks. XGBoost also outperformed traditional decision tree models in terms of training speed and accuracy, while still retaining good explanatory power [37]. Based on our evaluation of model performance, we consider XGBoost to be the most effective model for classifying the individual risk of CAD in patients with essential hypertension. Gupta *et al*. have applied ML in many areas including software maintenance [21, 32], smart homes [28] and medical tasks [24] with outstanding results [29]. Mittas *et al*. [8] made the first attempt at applying ML for CAD assessment. They excluded patients with coronary angiography results suggestive of non-CAD, and then proceeded to construct a deep learning model with a mean absolute error (MAE) of 5.6916. However, their model had a major limitation in that it excluded the non-diseased population upfront, thereby reducing the 0-factor interference [8]. It is important to note that application of ML algorithms in the medical field still faces multiple challenges and limitations. These include ensuring the transparency and interpretability of algorithms, as well as addressing data imbalance and privacy issues. Further research is necessary to overcome these obstacles and to advance the application of ML in the field of CAD diagnosis [38].

Exploratory and statistical analyses have shown that several risk factors for CAD are important for predicting whether patients have this disease [39]. In the present study, we provided objective evidence of risk factors that affect SYNTAX and GENSINI scores in the absence of knowledge about the relationship between SYNTAX scores and predictors [24].

Regarding future research on the application of machine learning in the diagnosis of coronary heart disease, insights can be gleaned from other fields of study. Yu *et al*. [40] explored the issue of disease causality inference by constructing a machine learning knowledge base to identify correlations among multiple diseases. Shamseddine *et al*. [41] proposed privacy-preserving federated learning models, providing novel ideas for developing machine learning models that protect patient privacy. Similarly, Wassan *et al*. [24] developed a solution to patient privacy concerns by utilizing federated machine learning to facilitate mobile collaborative development of standard prediction models, while storing all training data locally, thereby separating machine learning from data storage in the cloud to prevent privacy issues in medical data sharing. In the future, building a coronary heart disease knowledge base can aid in comprehending the linkages between coronary heart disease and multiple related illnesses. Furthermore, to mitigate the challenge of insufficient medical data for machine learning modeling due to patient privacy issues, adopting a federated learning approach may be worthwhile.

In summary, the main findings of this study concerned the diagnosis of CAD and evaluation of its severity. It is important to accurately predict whether a patient has CAD, since the clinical management of this condition involves ordering downstream investigations or coronary angiography procedures prior to hospitalization. Accurate identification of people without CAD would allow them to avoid coronary angiography and to receive other recommendations, such as improving their lifestyle and undergoing regular physical examination. The results of the feature selection algorithm identified some of the risk factors that contribute to variation in the distribution of SYNTAX and GENSINI scores.

The application of ML prediction models to cardiovascular disease has been evaluated previously in patients with ACS [42]. ML algorithms for CAD have been applied in some clinical settings, including (i) the prediction of CAD using clinical variables and an interdisciplinary approach; (ii) improving the detection of functional CAD using computational hemodynamics (e.g., FFR-based algorithms); and (iii) assessing the ability to automatically predict CAD based on myocardial perfusion imaging. Current clinical practice for patients with suspected CAD relies on invasive coronary angiography and the post hoc calculation of a score based on the coronary angiographic findings to guide further treatment.

There have been few comprehensive studies of CAD through the lens of ML [38]. In the clinical setting, the individual risk model established here and based on the XGBoost algorithm could be further developed into a supplementary diagnostic system. The model could be applied for screening CAD in the population and also to assist physicians in diagnosing CAD during outpatient visits. This could ultimately improve early detection and control, with a high degree of practicality and feasibility. The model could also provide a realistic approximation of the coronary load score to assess the complexity of CAD.

The present study has several limitations. The large number of patients with a coronary score of zero and the non-homogeneous data created difficulties for the modeling process due to limitation of the sample size. The distribution of patients with non-zero scores was not concentrated [43]. The risk stratification ML framework was developed to help clinicians identify patients with suspected coronary heart disease who should be referred for further examination, or who should undergo emergency surgery.

Further work is needed to optimize the model by using data from multicenter studies with large sample sizes. The model then needs to be validated in a prospective cohort and deployed into the community and clinic. In addition, multidisciplinary factors could be integrated into the model by using bioinformatic and pharmacogenomic analysis to extract other validated biomarkers such as specific genotypes. In brief, once validated using prospective external cohorts, the model established in this study could help clinicians to make decisions that are often still quite challenging. This will eventually ease the pressure on hospitals and doctors in the COVID-19 era and speed up the diagnosis and treatment process.

## 5. Conclusions

Machine learning models based on electronic medical records can effectively assess the severity of coronary heart disease and can identify a new set of new risk factors in the disease, and this study points to new research directions for future work.

## Data Availability

The datasets used and/or analyzed during the current study are available from the corresponding author on reasonable request.

## Abbreviations

CAD, coronary artery disease; ML, machine learning; ACS, acute coronary syndrome; PCI, percutaneous coronary intervention; CABG, coronary artery bypass graft; XGBoost, extreme gradient boosting; RF, random forest; MSE, mean squared error; MAE, mean absolute error; MAPE, mean absolute prediction error; R2, determination coefficient; SVM, Support Vector Machine; NB, Naive Bayes; LR, Logistic Regression; GBC, Gradient Boosting Classifier; Adaboots, Adaptive Boosting; K-Neighbors, K-Nearest Neighbors Regression; BMI, Body Mass Index; SBP, systolic blood pressure; DBP, diastolic blood pressure; TnI, Troponin I; CPK, creatine-phosphokinase; MB, myoglobin; BNP, brain natriuretic peptide; Glu, glucose; TG, Triglyceride; TC, Total cholesterol; HDL, high density lipoprotein; LDL, low-density lipoprotein; CRP, C Reactive Protein; IL6, Interleukin 6; CT, clotting time; Hcy, homocysteine; GHb, Glycosylated Hemoglobin; EF%, ejection fraction; LVWMAs, Left ventricular wall motion abnormalities; ARBs, angiotensin receptor blockers; CCBs, calcium channel blockers; FN, False Negative; TP, True Positive; TN, True Negative; FP, False Positive.

## Supplementary Material

Supplementary material associated with this article can be found, in the online version, at https://doi.org/10.31083/j.rcm2406168.
