# Supplementary material for: A Machine Learning Framework for Diagnosing and Predicting the Severity of Coronary Artery Disease
Source: Rev Cardiovasc Med. 2023 Jun 8;24(6):168. doi: 10.31083/j.rcm2406168 (PMC11264126; doi:10.31083/j.rcm2406168)
Supplement: Supplementary file 1 [file 2153-8174-24-6-168-s1.docx]

**Supplementary Table 1: Model introduction and parameter table.**

| Model | Introduction | Parameter Setting |
| --- | --- | --- |
| SVM | Support Vector Machine can map a dataset into a high-dimensional space and find an optimal hyperplane (i.e., decision boundary) in that space, which is used to distinguish different classes of data points. | kernel = 'poly', probability=True,C= 1 |
| XGB | eXtreme Gradient Boosting is an integrated machine learning model based on decision trees, which improves the performance of the model by iteratively adding weak classifiers. Each weak classifier is a decision tree, and the training data is used to continuously fit the underlying relationships and patterns of the data to obtain the final strong classifier. | n_estimators=312,use_label_encoder=False,eval_metric='mlogloss'，alpha=0.1，lambda=0.1，max_depth=3，scale_pos_weight=1/2 |
| RF | Random Forest is an integrated learning-based decision tree algorithm that reduces the risk of overfitting of individual decision trees and improves the overall prediction accuracy by building multiple decision trees. | n_estimators=100,n_jobs=1 |
| NB | Naive Bayes is a simple probabilistic classification algorithm based on Bayes' theorem, which calculates the probability of each category based on the given input features and their corresponding categories, and selects the category with the highest probability as the final prediction result. | GaussianNB |
| LR | Logistic Regression is a machine learning algorithm widely used in classification problems, which models the output results by constructing a linear function based on given input features. | Defaults |
| GBC | Gradient Boosting Classifier is an integrated decision tree-based learning algorithm that progressively improves the classification performance of the overall model by iteratively training weak classifiers, each time focusing on compensating for misclassified samples in the previous training round. | n_estimators=1000,learning_rate=0.1 |
| Adaboots | Adaboost (Adaptive Boosting) is an integrated learning algorithm for solving classification and regression problems. It forms a strong learner by combining multiple weak learners and improves the accuracy of the model by continuously adjusting the sample weights.。 | max_depth=2, min_samples_split=20, min_samples_leaf=5 |
| Decision Tree | Decision Tree is a model based on a tree structure for decision analysis and is used to solve classification and regression problems. The decision tree model divides the data set into many subsets, each of which is given a label, and then constructs a tree structure that starts at the root node and splits recursively by feature attributes up to the leaf nodes. | criterion = entropy，min_samples_split=10 |
| linear | Linear Regression is a model used to establish a linear relationship between the independent and dependent variables, which assumes a linear relationship between the independent and dependent variables and tries to find an optimal straight line that minimizes the mean error (sum of squared residuals) between the predicted and actual values. | copy_X= false，alpha=0.1 |
| K-Neighbors | K-Nearest Neighbors Regression is a non-parametric regression model based on instance learning by finding the K nearest neighbors in the training set and using the average of their outputs (target values) as the predicted output. | n_neighbors=10 |
| Bagging | Bagging regression model is an integrated learning approach, also known as bootstrap aggregating, which builds multiple base models by sampling different training datasets and averaging or voting their outputs to get the final prediction. | n_estimators =10 |
| Extra-Tree | Extra-Tree regression model is also known as Extreme Randomized Trees Regression. The Extra-Tree model uses more randomness and optimization strategies in constructing the tree to improve the accuracy and generalization ability of the model. | n_estimators=10，max_depth=3，bootstrap =true |

**Supplementary Figure1: 5-fold cross-validation strategy.**

| **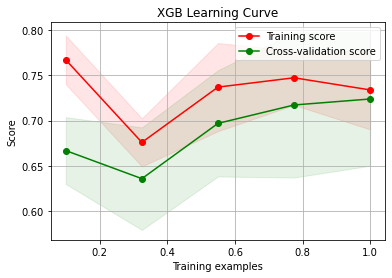** | **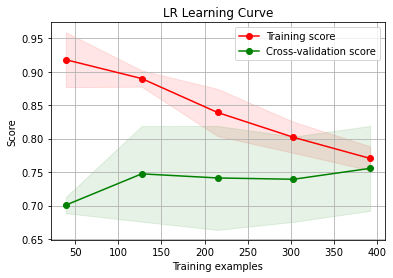** | **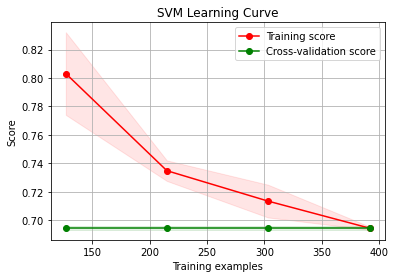** |
| --- | --- | --- |

we incorporated a 5-fold cross-validation strategy in the methodology section to optimize model performance. Additionally, we employed grid search to fine-tune the hyperparameters of the model during the 5-fold cross-validation process. Specifically, we specified a set of potential hyperparameters and constructed a parameter space by combining them. Subsequently, we assessed the model performance using all possible hyperparameter combinations and selected the combination with the highest average test performance as the optimal set of hyperparameters.
